# Associations of Night Eating Behavior and Body Mass Index with Sleep Quality in Adult Women: A Cross-Sectional Study

**DOI:** 10.3390/nu18213655

**Published:** 2026-11-03

**Authors:** Özlem Baran, Cemaliye Süt Kurt, Muhammed Enes Kartal, Birgül Dağ, Ayhan Dağ, Sevinç Yücecan, Hilmiye Nesrin Dilbaz

**Affiliations:** 1 Department of Nutrition and Dietetics, Faculty of Health Sciences, Lokman Hekim University, Ankara 06510, Türkiye; muhammed.kartal@lokmanhekim.edu.tr (M.E.K.); birgul.dag@lokmanhekim.edu.tr (B.D.); ayhan.dag@lokmanhekim.edu.tr (A.D.); sevinc.yucecan@lokmanhekim.edu.tr (S.Y.); 2 Department of Nutrition and Dietetics, Faculty of Health Sciences, Cyprus International University, Nicosia 99258, Cyprus; dyt.cemaliyesut@gmail.com; 3 Department of Psychiatry, Faculty of Medicine, Üsküdar University, Istanbul 34662, Türkiye; nesrin.dilbaz@gmail.com

**Keywords:** sleep quality, night eating behavior, body mass index, Pittsburgh Sleep Quality Index, Night Eating Questionnaire

## Abstract

Background: Night eating behavior, adiposity, and poor sleep quality have been examined in previous studies, but additional evidence from large female samples with directly measured anthropometric characteristics is valuable. This study aimed to examine the associations among night eating behavior, body mass index (BMI), and subjective sleep quality in adult women, including their associations after adjustment for sociodemographic and lifestyle factors. Methods: This analytical cross-sectional study included 1203 women aged 18–65 years recruited from four family centers in Mamak, Ankara, Türkiye. Night eating behavior was assessed using the Night Eating Questionnaire (NEQ), and subjective sleep quality was evaluated using the Pittsburgh Sleep Quality Index (PSQI). Anthropometric measurements were obtained directly by the researcher. Correlation analyses and hierarchical multiple linear regression analyses were performed, with adjustment for age, education, occupation, smoking, alcohol consumption, and regular exercise. Results: The mean PSQI and NEQ total scores were 5.58 ± 2.84 and 12.50 ± 5.04, respectively. Night eating behavior was moderately positively correlated with PSQI scores (ρ = 0.404, *p* < 0.001), indicating poorer subjective sleep quality at higher NEQ scores. BMI showed weak positive correlations with PSQI scores (r = 0.122, *p* < 0.001) and NEQ scores (ρ = 0.085, *p* = 0.003). In the fully adjusted regression model, NEQ remained significantly associated with higher PSQI scores (β = 0.425, *p* < 0.001), whereas BMI was not significantly associated with PSQI scores (β = 0.051, *p* = 0.115). Older age and current smoking were associated with higher PSQI scores, while regular exercise was associated with lower scores. The final model explained 22.9% of the variance in PSQI scores (adjusted R^2^ = 0.218). Conclusions: Night eating behavior was moderately associated with poorer subjective sleep quality and remained significantly associated with PSQI scores after adjustment for measured sociodemographic and lifestyle factors. Although BMI showed weak unadjusted associations with sleep quality and night eating behavior, its association with sleep quality was not statistically significant in the fully adjusted model. These findings highlight the relevance of night eating-related symptoms to subjective sleep quality in adult women, without establishing a causal relationship.

## 1. Introduction

Sleep is a fundamental biological process essential for maintaining life and preserving optimal health. It plays a critical role in regulating numerous physiological processes, including metabolism, appetite regulation, immune function, hormonal homeostasis, and cardiovascular function [1]. Insufficient or poor-quality sleep has been associated with alterations in neuroendocrine stress responses, autonomic nervous system dysregulation, and metabolic disturbances. In modern societies, lifestyle changes, shift work, and voluntary sleep restriction have substantially altered both sleep duration and sleep quality [2].

Short sleep duration and poor sleep quality have been consistently associated with cardiometabolic risk factors, including obesity, type 2 diabetes, and cardiovascular disease [3]. One of the most widely proposed mechanisms underlying these associations involves alterations in appetite-regulating hormones. Sleep deprivation has been shown to decrease circulating leptin levels, an appetite-suppressing hormone, while increasing circulating ghrelin levels, an appetite-stimulating hormone [4]. These hormonal alterations may increase hunger, promote greater energy intake, and contribute to positive energy balance, ultimately leading to weight gain [5]. Furthermore, epidemiological evidence indicates that shorter sleep duration is associated with higher total energy intake, increased dietary fat consumption, and poorer overall diet quality [6].

In recent years, late-night eating has emerged as an important behavioral risk factor that adversely affects metabolic health. Alterations in the timing of the sleep–wake cycle may lead to misalignment between behavioral patterns and endogenous circadian physiology [7,8]. Such circadian misalignment has been shown to disrupt energy metabolism and impair metabolic regulation [9,10]. Furthermore, it may adversely affect glucose metabolism and increase cardiovascular risk factors [11,12].

Food consumption during the habitual sleep period, together with increased energy intake and irregular eating patterns, has been suggested to contribute to night eating behavior and obesity [13]. Previous studies have examined the relationships among night eating behavior, sleep quality, and adiposity, including investigations conducted specifically among women [14]. Recent studies have further explored these associations in different populations. A 2025 study among medical students investigated the relationships between night eating symptoms, sleep-related factors, and BMI [15] while a 2026 study among Saudi Arabian women examined night eating behavior, sleep quality, and body composition [16]. Additionally, a 2026 study conducted among adults in Türkiye reported a positive association between night eating behavior and poor sleep quality [17]. However, differences in study populations, recruitment settings, anthropometric assessment methods, and statistical approaches may influence the interpretation of these associations. Further evidence from large samples of adult women recruited from family centers, with directly measured anthropometric characteristics, may therefore complement existing findings.

Against this background, understanding the relationship between sleep characteristics and eating behaviors has become increasingly important, particularly in the context of body weight management [18]. The present study aimed to examine the associations among night eating behavior, body mass index (BMI), and subjective sleep quality in a large sample of adult women recruited from family centers. By incorporating directly measured anthropometric characteristics and multivariable analyses accounting for sociodemographic and lifestyle factors, this study sought to provide additional evidence regarding these relationships in a community-based female population. We hypothesized that night eating behavior and BMI would be associated with subjective sleep quality, although the magnitude and independence of these associations after adjustment for potential confounding factors remained uncertain.

## 2. Materials and Methods

### 2.1. Study Design and Participants

This analytical cross-sectional study was conducted among adult women aged 18–65 years between June and July 2024. Participants were recruited using a convenience sampling approach from women attending four family health centers in the Mamak district of Ankara, Türkiye. All women attending these centers during the data collection period were assessed for eligibility according to the study’s inclusion criteria. Women who met the inclusion criteria, voluntarily agreed to participate, and provided written informed consent were enrolled in the study. The sample size was recalculated using G*Power 3.1 with the F-test family and the “Linear multiple regression: Fixed model, R^2^ deviation from zero” option. The analysis used an effect size of f^2^ = 0.02, α = 0.05, 95% statistical power, and two predictors (NEQ and BMI). The minimum required sample size was 776 participants. A total of 1203 women were included in the study, exceeding this requirement. The exclusion criteria were male sex, age below 18 or above 65 years, pregnancy, a diagnosed psychiatric disorder, a diagnosed sleep disorder, and unwillingness to participate in the study. The study protocol was approved by the Non-Interventional Clinical Research Ethics Committee of Lokman Hekim University (Approval No. 2024/99). All study procedures were conducted in accordance with the principles of the Declaration of Helsinki, and written informed consent was obtained from all participants prior to enrollment.

Data were collected using a structured questionnaire that included sociodemographic characteristics. Night eating behavior was assessed using the Night Eating Questionnaire (NEQ), and sleep quality was evaluated using the Pittsburgh Sleep Quality Index (PSQI). All data were collected through face-to-face interviews conducted by the researcher. Exercise habits were assessed through self-reported questions regarding regular exercise participation, frequency, and duration. For statistical analyses, regular exercise was categorized based on participants’ yes/no responses, without applying a predefined frequency or duration threshold.

Of the 1203 participants, NEQ data were missing for one participant. Accordingly, analyses involving NEQ, including the regression models, were conducted using complete cases (*n* = 1202), with no imputation. The PSQI–BMI correlation was based on all 1203 participants, for whom data on both variables were available. Pairwise deletion was used for the correlation analyses, whereas regression analyses were conducted using complete cases for the variables included in each model.

### 2.2. Pittsburgh Sleep Quality Index (PSQI)

Sleep quality was assessed using the Pittsburgh Sleep Quality Index (PSQI), originally developed by Buysse et al. to evaluate subjective sleep quality over the previous month [19]. The Turkish version of the PSQI was validated by Ağargün et al. and demonstrated acceptable reliability for use in the Turkish population [20]. The reported Cronbach’s alpha coefficients were 0.83 for the original version and 0.80 for the Turkish version.

The PSQI consists of 24 items, including 19 self-rated items that contribute to the global score and five items completed by a roommate or bed partner that are not included in the scoring. The questionnaire comprises seven component scores: subjective sleep quality, sleep latency, sleep duration, habitual sleep efficiency, sleep disturbances, use of sleep medication, and daytime dysfunction. The global PSQI score ranges from 0 to 21, with scores ≤5 indicating good sleep quality and scores >5 indicating poor sleep quality. In the present study, only the global PSQI score was used for statistical analyses.

### 2.3. The Night Eating Questionnaire (NEQ)

Night eating behavior was assessed using the Night Eating Questionnaire (NEQ), originally developed by Allison et al. to evaluate the presence and severity of night eating symptoms [21]. The Turkish version of the NEQ was validated by Atasoy et al. and demonstrated acceptable internal consistency (Cronbach’s α = 0.69) [22]. The questionnaire consists of 14 items rated on a five-point Likert scale, yielding a total score ranging from 0 to 52, with higher scores indicating a greater tendency toward night eating behavior. A total score of ≥25 is generally considered indicative of a clinically significant risk of night eating syndrome. In the present study, the total NEQ score was used to assess the tendency toward night eating behavior rather than to establish a clinical diagnosis of night eating syndrome.

### 2.4. Anthropometric Measurements

Participants’ body weight was measured using a portable scale with a precision of 0.1 kg, while wearing light clothing and no shoes. Height was measured using a stadiometer with a headpiece, with a precision of 0.1 cm. Participants stood barefoot with their feet together and their back, buttocks, and heels touching the wall, with their heads positioned in the Frankfurt horizontal plane, defined by the alignment of the inferior margin of the orbit and the superior margin of the external auditory meatus in the same horizontal plane. Height was measured during deep inspiration. Body mass index (BMI) was calculated as body weight (kg) divided by the square of height (m^2^).

Waist circumference was measured at the midpoint between the lowest rib and the iliac crest using a non-elastic measuring tape. Hip circumference was measured using the same non-elastic measuring tape at the widest point below the iliac crest and over the buttocks. Both measurements were recorded with a precision of 0.1 cm. Waist-to-hip ratio was calculated by dividing waist circumference (cm) by hip circumference (cm), while waist-to-height ratio was calculated by dividing waist circumference (cm) by height (cm). Anthropometric measurements were performed once for each participant.

### 2.5. Statistical Analysis

Statistical analyses were performed using IBM SPSS Statistics software (version 29.0; IBM Corp., Armonk, NY, USA). Continuous variables were summarized as mean ± standard deviation (SD) and median (minimum–maximum), while categorical variables were presented as frequencies (n) and percentages (%). The distribution of continuous variables was assessed using skewness and kurtosis values, supplemented by visual inspection of histograms and quantile–quantile (Q–Q) plots. Skewness and kurtosis values within ±2 were considered heuristic indicators of approximate normality rather than definitive criteria for selecting statistical tests. BMI and PSQI total scores met this criterion, although their distributions exhibited mild right-skewness, whereas NEQ total scores showed greater deviation from normality. Regression residuals were evaluated separately using histograms and Q–Q plots.

Independent-samples *t*-tests were used to compare approximately normally distributed continuous variables between two independent groups. Homogeneity of variances was assessed using Levene’s test, and Welch’s *t*-test was applied when the assumption of equal variances was violated. The Mann–Whitney U test was used to compare NEQ total scores between participants classified as having good and poor sleep quality. The effect size for this comparison was calculated as r = Z/√N, where Z represents the standardized test statistic and N represents the total number of observations included in the comparison. Pearson’s correlation coefficient (r) was used to assess associations between approximately normally distributed continuous variables, whereas Spearman’s rank correlation coefficient (ρ) was used when at least one variable deviated from normality. The 95% confidence intervals (CIs) for correlation coefficients were estimated using 3000 bootstrap resamples.

Hierarchical multiple linear regression analysis was conducted to examine the associations of night eating behavior and BMI with subjective sleep quality after accounting for sociodemographic and lifestyle characteristics. The global PSQI score was specified as the dependent variable. Predictors were entered sequentially into three models. Model 1 included the NEQ total score; Model 2 additionally included BMI; and Model 3 further included age, educational status, occupational status, smoking status, alcohol consumption, and regular exercise. Changes in explained variance (ΔR^2^) and F-change statistics were used to evaluate the incremental contribution of predictors entered at successive steps. Model fit was evaluated using R^2^, adjusted R^2^, and the overall F statistic.

To account for potential heteroscedasticity, heteroscedasticity-consistent standard errors were estimated using the HC3 method in the fully adjusted regression model. Regression coefficients were reported as unstandardized coefficients (B), standardized coefficients (β), and corresponding 95% CIs and *p*-values. Multicollinearity was assessed using variance inflation factors (VIF) and tolerance values, with VIF values below 10 and tolerance values above 0.10 used as screening criteria.

Potential outlying and influential observations were examined using externally studentized residuals and Cook’s distance. Observations with absolute externally studentized residuals greater than 3 were identified for further assessment. A sensitivity analysis was performed by refitting the fully adjusted regression model after excluding these observations to evaluate whether the main associations were materially affected. All observations with complete data were retained in the primary regression analysis. To assess the potential influence of overlapping sleep-related content between the NEQ and PSQI, an exploratory sensitivity analysis was conducted. A modified NEQ score was calculated by excluding items 8, 9, and 11, which assess difficulty falling asleep, nocturnal awakenings, and the need to eat to return to sleep, respectively. The association between the modified NEQ score and the global PSQI score was examined using Spearman’s correlation analysis. In addition, the hierarchical regression models were repeated using the modified NEQ score instead of the original NEQ total score, while retaining the same covariates. The modified NEQ score was used solely for this exploratory sensitivity analysis and was not considered a validated alternative to the original NEQ total score.

Of the 1203 participants, one had missing NEQ data. Pairwise deletion was applied in correlation analyses, whereas complete-case analysis was used for the regression models and other analyses requiring NEQ scores (*n* = 1202). No missing values were imputed. The internal consistency of the NEQ and PSQI was assessed using Cronbach’s alpha coefficients, calculated from the 13 scored NEQ items and the seven PSQI component scores, respectively. All statistical tests were two-sided, and *p*-values below 0.05 were considered statistically significant.

## 3. Results

As shown in Table 1, the mean body weight of the participants was 70.26 ± 14.10 kg, and the mean BMI was 27.32 ± 5.63 kg/m^2^. The mean global PSQI total score was 5.58 ± 2.84, while the mean Night Eating Questionnaire (NEQ) total score was 12.50 ± 5.04.

Table 2 presents the distribution of the participants according to their sociodemographic characteristics and lifestyle behaviors. Of the participants, 78.2% were married and 21.8% were single. Regarding educational level, 37.0% had completed high school, 30.1% had completed primary/secondary school, and 27.1% had a university degree. The proportions of illiterate participants and those with a postgraduate (Master’s/PhD) degree were 2.7% and 3.2%, respectively.

With respect to occupation, the majority of participants were housewives/unemployed (64.9%), followed by civil servants (10.6%) and private sector employees (9.8%). Students, self-employed individuals, and retirees accounted for 5.6%, 6.2%, and 2.7% of the study population, respectively. Participants in the “other” occupational category represented 0.2% of the sample. Regarding smoking status, 71.5% of the participants were non-smokers, 25.6% were current smokers, and 2.9% were former smokers. The majority of participants did not consume alcohol (91.2%), whereas 8.4% reported current alcohol consumption and 0.4% reported having quit alcohol consumption. In terms of regular physical activity, 25.3% of the participants reported engaging in regular exercise, whereas 74.7% reported not exercising regularly.

Cronbach’s alpha was 0.515 for the 13 scored NEQ items (*n* = 1202), indicating low internal consistency in the present sample. For the seven PSQI component scores, Cronbach’s alpha was 0.540 (*n* = 1203), also indicating low internal consistency.

Correlation analyses showed a moderate positive association between NEQ and PSQI total scores (Spearman’s ρ = 0.404, 95% CI: 0.355–0.454, *p* < 0.001). Higher NEQ scores were associated with higher PSQI scores, indicating poorer subjective sleep quality. NEQ total score was weakly positively correlated with BMI (Spearman’s ρ = 0.085, 95% CI: 0.027–0.140, *p* = 0.003). BMI was also weakly positively correlated with PSQI total score (Pearson’s *r* = 0.122, 95% CI: 0.065–0.179, *p* < 0.001). Although all correlations were statistically significant, those involving BMI were small in magnitude. Analyses involving NEQ included 1202 participants; the PSQI–BMI correlation included 1203 participants (Table 3).

Hierarchical multiple linear regression analysis was conducted using complete cases. Of the 1203 participants in the dataset, one participant with a missing NEQ total score was excluded through listwise deletion; therefore, the regression models included 1202 participants. In the first step, NEQ total score was entered, and the model explained 18.6% of the variance in PSQI scores (R^2^ = 0.186; F(1, 1200) = 274.181, *p* < 0.001). Adding BMI in the second step increased the explained variance by 0.9% (ΔR^2^ = 0.009; ΔF(1, 1199) = 13.631, *p* < 0.001). In the final step, age, education, occupation, smoking status, alcohol use, and regular exercise were added, accounting for an additional 3.3% of the variance (ΔR^2^ = 0.033; ΔF(15, 1184) = 3.426, *p* < 0.001). The final model was statistically significant and explained 22.9% of the variance in PSQI scores (R^2^ = 0.229; adjusted R^2^ = 0.218; F(17, 1184) = 20.642, *p* < 0.001). In the fully adjusted model, higher NEQ scores (B = 0.240, 95% CI: 0.208 to 0.272, *p* < 0.001), older age (B = 0.031, 95% CI: 0.015 to 0.047, *p* < 0.001), and current smoking, compared with no smoking (B = 0.563, 95% CI: 0.191 to 0.936, *p* = 0.003), were associated with higher PSQI scores, indicating poorer subjective sleep quality. Regular exercise was associated with lower PSQI scores (B = −0.397, 95% CI: −0.721 to −0.074, *p* = 0.016). BMI was not significantly associated with PSQI scores after adjustment (B = 0.026, 95% CI: −0.006 to 0.058, *p* = 0.115) (Table 4 and Table 5).

Four observations had absolute externally studentized residuals greater than 3. The maximum Cook’s distance was 0.030. A sensitivity analysis excluding these four observations did not materially change the main findings: NEQ remained associated with higher PSQI scores (*B* = 0.240, *p* < 0.001), whereas the association between BMI and PSQI remained nonsignificant (*B* = 0.021, *p* = 0.160). All observations were retained in the primary analysis.

Participants classified as having poor sleep quality (PSQI > 5) had higher mean body weight than those classified as having good sleep quality (PSQI ≤ 5; 71.63 ± 15.20 vs. 69.15 ± 13.04 kg, *p* = 0.003). The poor sleep quality group also had higher mean BMI (28.03 ± 6.06 vs. 26.76 ± 5.19 kg/m^2^, *p* < 0.001), waist circumference (89.68 ± 15.12 vs. 86.18 ± 13.45 cm, *p* < 0.001), hip circumference (107.46 ± 11.89 vs. 105.25 ± 11.50 cm, *p* = 0.001), waist-to-hip ratio (0.82 ± 0.09 vs. 0.81 ± 0.08, *p* = 0.006), and waist-to-height ratio (0.55 ± 0.10 vs. 0.53 ± 0.08, *p* < 0.001). Conversely, mean height was lower in the poor sleep quality group (160.07 ± 6.32 vs. 160.92 ± 6.23 cm, *p* = 0.020) (Table 6).

Among participants with complete NEQ data, women classified as having poor sleep quality (PSQI > 5; *n* = 539) had higher NEQ scores than those classified as having good sleep quality (PSQI ≤ 5; *n* = 663). The median NEQ score was 13 [Q1–Q3: 11–17] in the poor-sleep group and 11 [Q1–Q3: 8–13] in the good-sleep group; the corresponding mean ranks were 728.06 and 498.61, respectively. This between-group difference was statistically significant (Mann–Whitney *U* = 110,461.5, *Z* = −11.427, *p* < 0.001), with a moderate effect size (*|r|* = 0.330). One participant in the poor-sleep group had missing NEQ data and was excluded from this comparison (Table 7).

An exploratory sensitivity analysis was conducted by excluding three sleep-related items (8, 9, and 11) from the NEQ. The correlation between the modified NEQ score and the global PSQI score was weaker but remained statistically significant (Spearman’s ρ = 0.230, *p* < 0.001, *n* = 1202), compared with the original NEQ score (ρ = 0.404, *p* < 0.001). In the hierarchical regression analysis using the modified NEQ score, the explained variance was 6.8% in Model 1 (R^2^ = 0.068), 7.9% after adding BMI in Model 2 (R^2^ = 0.079), and 11.8% in the fully adjusted Model 3 (R^2^ = 0.118; adjusted R^2^ = 0.105), compared with 18.6%, 19.5%, and 22.9%, respectively, in the original models. The modified NEQ score remained significantly associated with higher PSQI scores in the fully adjusted model (B = 0.201, β = 0.257, 95% CI: 0.158–0.244, *p* < 0.001). These findings indicate that the association persisted after excluding sleep-related NEQ items, although its magnitude was attenuated.

## 4. Discussion

Poor sleep quality and night eating behavior have recently attracted considerable attention because of their associations with metabolic, circadian, and behavioral health. However, studies simultaneously evaluating these two variables together with body mass index (BMI) remain limited. The aim of the present study was to evaluate the associations among sleep quality, night eating behavior, and BMI in adult women and to examine whether night eating behavior and BMI were independently associated with subjective sleep quality after adjustment for sociodemographic and lifestyle characteristics.

The main findings of the present study demonstrated a moderate positive association between night eating behavior and PSQI scores, indicating that higher night eating scores were associated with poorer subjective sleep quality. Significant but weak positive correlations were also observed between BMI and PSQI scores and between night eating behavior and BMI. Importantly, in the fully adjusted regression model, night eating behavior remained significantly associated with poorer sleep quality, whereas the association between BMI and PSQI scores was no longer statistically significant. In addition, older age and current smoking were associated with higher PSQI scores, while regular exercise was associated with lower PSQI scores.

Regarding the general characteristics of the study population, the mean global PSQI score among the 1203 participants was 5.58 ± 2.84, and 44.8% (*n* = 540) of the participants were classified as having poor sleep quality. The mean BMI of the participants (27.32 ± 5.63 kg/m^2^) was within the overweight range, suggesting that excess body weight was a relevant characteristic of the study population. Although the mean NEQ total score (12.50 ± 5.04) was below the commonly used screening threshold of 25, this finding alone does not establish the presence or absence of Night Eating Syndrome (NES) at the individual level. It is important to distinguish night eating behavior from NES, late eating, and circadian eating misalignment. Night eating behavior, as assessed by the NEQ, reflects a range of night eating-related symptoms, whereas NES is a clinical condition characterized by evening hyperphagia and/or nocturnal ingestions accompanied by additional diagnostic features. Late eating refers primarily to the timing of food consumption, while circadian eating misalignment involves a mismatch between eating patterns and the endogenous circadian system. Although these concepts are related, they are not interchangeable. In the present study, NEQ scores were used to assess night eating-related symptoms rather than to establish a clinical diagnosis of NES or to directly measure meal timing or circadian misalignment. Higher NEQ scores were associated with poorer sleep quality in the present sample. This finding is consistent with previous research reporting associations between night eating-related behaviors and sleep disturbances. Yeh and Brown [23] reported a significant association between poor sleep quality and night eating behavior. Crispim et al. [24] found that late-night food intake was associated with lower sleep efficiency and prolonged sleep latency, whereas Griffith et al. [25] reported that lower nighttime food intake was associated with optimal sleep efficiency. Similarly, several studies have reported associations between impaired sleep quality and night eating-related or circadian eating patterns [26,27,28]. However, these findings should be compared cautiously because the studies assessed related but distinct aspects of eating behavior and timing. In addition, the relatively low internal consistency of the NEQ (Cronbach’s α = 0.515) and PSQI (Cronbach’s α = 0.540) in the present sample should be considered when interpreting this association. These findings may be affected by measurement error, and the observed correlation may not fully represent the relationship between the underlying constructs. Furthermore, the exploratory sensitivity analysis provided additional insight into the potential influence of conceptual overlap between the NEQ and PSQI. After excluding three sleep-related NEQ items, the correlation with PSQI scores decreased from ρ = 0.404 to ρ = 0.230 but remained statistically significant. Similarly, the explained variance in the fully adjusted regression model decreased from 22.9% to 11.8%, although the modified NEQ score remained significantly associated with PSQI scores. These findings suggest that overlapping sleep-related content may have contributed to the magnitude of the original association. Nevertheless, the persistence of a weaker association after excluding these items suggests that the observed relationship may not be explained entirely by overlapping sleep-related questionnaire content. However, given the exploratory nature of the modified NEQ score and the relatively low internal consistency of the original instruments, these findings should be interpreted cautiously.

Although the cross-sectional design precludes causal interpretations, several circadian and physiological mechanisms may contribute to the observed association between night eating-related symptoms and subjective sleep quality. Previous research has suggested that eating during the biological night may be associated with circadian disruption and alterations in melatonin-related processes [29]. Changes in stress-related neuroendocrine activity, including hypothalamic–pituitary–adrenal (HPA) axis regulation, may also be relevant to the relationship between eating patterns and sleep disturbances [30]. Furthermore, the thermogenic effects of food intake may influence the nocturnal decline in core body temperature, potentially affecting sleep initiation and continuity [31,32]. However, these mechanisms were not directly assessed in the present study, and the NEQ does not provide a direct measure of meal timing or circadian phase.

In the present study, BMI showed a weak but statistically significant positive correlation with PSQI scores, indicating that higher BMI was associated with poorer subjective sleep quality in the unadjusted analysis. This finding is consistent with previous meta-analyses [33] and studies conducted among women [34], as well as other investigations reporting associations between poor sleep quality and obesity-related outcomes [35,36]. A recent study among children and adolescents also examined the association between sleep duration and overweight/obesity, although differences in age group and sleep-related measures limit direct comparisons with the present findings [37]. However, unlike the unadjusted correlation, the association between BMI and PSQI scores was no longer statistically significant after adjustment for age, education, occupation, smoking status, alcohol use, and regular exercise (β = 0.051, *p* = 0.115). This finding suggests that the observed relationship between BMI and subjective sleep quality may partly reflect differences in sociodemographic and lifestyle characteristics. In this respect, the results of the fully adjusted model are more consistent with studies reporting no significant association between BMI and sleep quality [38,39]. Nevertheless, differences in study populations, sleep assessment methods, and statistical adjustments may contribute to the inconsistent findings across studies. Several biological mechanisms have been proposed to explain the relationship between sleep disturbances and obesity. Poor sleep quality may be associated with alterations in hypothalamic–pituitary–adrenal (HPA) axis activity, appetite-regulating hormones such as ghrelin and leptin, and insulin sensitivity [4,40,41]. In addition, obesity-related conditions, including obstructive sleep apnea, may contribute to impaired sleep quality [42]. However, these mechanisms were not directly investigated in the present study and cannot explain the observed associations with certainty. Importantly, night eating behavior remained significantly associated with PSQI scores in the fully adjusted regression model, whereas BMI did not. This finding indicates that the association between night eating behavior and subjective sleep quality was more robust to adjustment for the measured sociodemographic and lifestyle characteristics. Nevertheless, this does not establish the relative importance of behavioral and metabolic mechanisms, as BMI is an anthropometric index and metabolic biomarkers were not assessed. Furthermore, BMI does not distinguish between fat mass and lean mass and may not adequately reflect individual differences in adiposity. A study by Falbová et al. among Slovak young adults identified individuals with excess body fat despite having a BMI in the normal range, illustrating the limitations of BMI as a standalone indicator of adiposity. Although the study did not examine the association between night eating symptoms and sleep quality, it provides contextual support for considering body composition alongside BMI [43].

In the unadjusted correlation analysis, a statistically significant but very weak positive association was observed between night eating behavior and BMI (ρ = 0.085, *p* = 0.003). Consistent with our findings, several previous studies have reported a positive association between night eating behavior and BMI [15,44,45], whereas others have found no significant association [46,47]. These discrepancies may be attributable to differences in sample size or the sociodemographic characteristics of the study populations. The association between night eating-related symptoms and BMI may involve complex interactions among circadian regulation, eating behaviors, and metabolic processes. Alterations in insulin sensitivity and appetite-regulating hormones, including leptin and ghrelin, have been proposed as potential mechanisms linking eating patterns and body weight [48]. However, these mechanisms were not directly assessed in the present study, and the very weak correlation observed suggests that the association was limited in magnitude. A study by Sulis et al. [49] examining sex-specific relationships among sleep routines, nutritional habits, and somatic health indicators in young adults provides further context for these findings. Among women, irregular sleep routines and late-night eating habits were associated with higher BMI, suggesting that sleep-related and dietary behaviors may be interconnected with anthropometric characteristics. This finding is broadly consistent with the weak positive association between night eating behavior and BMI observed in the present study. However, important methodological differences should be considered. Sulis et al. assessed sleep routines and the frequency of late-night eating, whereas the present study evaluated night eating-related symptoms using the NEQ and subjective sleep quality using the PSQI. Furthermore, their sample consisted of young adults, while our study included women across a wider age range. Therefore, the findings should be interpreted as complementary evidence rather than directly comparable associations.

Only a limited number of studies have simultaneously evaluated night eating behavior, sleep quality, and BMI. Yeh et al. [23] investigated the relationship among night eating behavior, sleep quality, and BMI and, unlike the present study, also assessed binge eating disorder. Similar to our findings, Yeh et al. reported a significant association between poor sleep quality and night eating behavior. They also found that higher BMI was associated with adverse sleep characteristics, including shorter sleep duration, longer sleep latency, and the use of sleep medication. While sleep was evaluated in greater detail using individual PSQI component scores in their study, the present study used the global PSQI score. Furthermore, although Yeh et al. observed a correlation between BMI and night eating behavior, this association did not remain statistically significant in their multiple regression analysis. In the present study, BMI and night eating behavior were also weakly but significantly correlated. However, when PSQI scores were examined as the dependent variable in the fully adjusted regression model, BMI was no longer significantly associated with sleep quality, whereas night eating behavior remained significantly associated with PSQI scores. Unlike Yeh et al., the present study did not investigate potential mediating relationships among BMI, night eating behavior, and sleep quality. Nevertheless, our findings underscore the importance of distinguishing unadjusted correlations from associations observed after multivariable adjustment. Given the cross-sectional design, no conclusions can be drawn regarding the direction or underlying mechanisms of these relationships.

In the fully adjusted regression model, age, smoking status, and regular exercise were also significantly associated with subjective sleep quality. Older age and current smoking were associated with higher PSQI scores, indicating poorer sleep quality, whereas regular exercise was associated with lower PSQI scores. These findings are consistent with previous research highlighting the relevance of demographic and lifestyle characteristics to sleep quality. A 12-year longitudinal cohort study reported age-related deterioration in sleep quality, with more pronounced worsening of PSQI scores among women [50]. Similarly, a large population-based study conducted in South Korea found that current smoking was significantly associated with poor sleep quality among women younger than 65 years, even after adjustment for psychological and other potential confounding factors [51]. Regarding physical activity, a recent systematic review and meta-analysis of randomized controlled trials demonstrated that exercise interventions significantly reduced PSQI scores in adult women [52]. Although differences in study design and population characteristics should be considered, these findings broadly support the associations observed in the present study and underscore the importance of accounting for sociodemographic and lifestyle characteristics when examining the relationship between night eating behavior and subjective sleep quality.

In addition, anthropometric measurements and NEQ scores were examined according to PSQI classification (good sleep quality vs. poor sleep quality). Participants with poor sleep quality had significantly higher body weight, BMI, waist circumference, hip circumference, waist-to-hip ratio, and waist-to-height ratio than those with good sleep quality. Mean height was also slightly lower in the poor sleep quality group, although the magnitude of this difference was small. These findings are consistent with a recent study among young adults reporting significant positive associations between PSQI scores and BMI, waist circumference, and waist-to-hip ratio [53]. However, findings in the literature are not entirely consistent. Suna and Ayaz reported significant differences in BMI and waist-to-hip ratio according to sleep quality only among male university students, despite a predominantly female sample [54]. Similarly, a recent study among adults with hypertension found that the association between BMI and poor sleep quality was significant in univariate analysis but not after multivariable adjustment [55]. These findings highlight the importance of distinguishing unadjusted anthropometric differences from independent associations. In the present study, the observed group differences should not be interpreted as independent associations, particularly because BMI was no longer significantly associated with PSQI scores in the fully adjusted regression model. Furthermore, participants with poor sleep quality had significantly higher NEQ scores, consistent with the correlation and regression findings. Supporting this result, Köse et al. reported significantly higher NEQ scores among female university students with poor sleep quality (*p* = 0.003) [56].

The findings of the present study suggest that night eating behavior may be relevant when assessing sleep quality in adult women. However, given the cross-sectional design, it remains unclear whether modifying night eating behavior would lead to improvements in sleep quality. Therefore, longitudinal and interventional studies are needed to clarify the direction of this association and to evaluate whether nutritional education, behavioral interventions, or lifestyle modifications targeting circadian rhythms could contribute to improved sleep quality. Because the study included only women, the findings should be interpreted within this population and should not be generalized to men. Female-specific factors, including hormonal and reproductive characteristics, may also be relevant to sleep and eating behaviors; however, these factors were not directly evaluated in the present study.

This study has several limitations. First, the cross-sectional design does not allow causal inferences to be made regarding the relationships between the variables. However, the findings may contribute to the development of theoretical models that could guide the design of future longitudinal studies. In addition, the assessment of sleep quality and night eating behavior using self-reported questionnaires may have introduced measurement bias. The internal consistency coefficient for the NEQ was low in the present sample (Cronbach’s α = 0.515), raising concerns about measurement reliability and warranting caution when interpreting associations involving NEQ total scores. Cronbach’s alpha calculated from the seven PSQI component scores was also relatively low (α = 0.540). However, because the PSQI comprises distinct dimensions of sleep quality, this coefficient should not be interpreted as a definitive assessment of the reliability of the global PSQI score. These measurement considerations should be taken into account when interpreting the findings. The relatively low internal consistency of both instruments may have introduced measurement error and reduced the precision of the observed associations. Therefore, the relationship between night eating behavior and subjective sleep quality should be interpreted cautiously, as the observed correlation may not fully reflect the association between the underlying constructs. Importantly, both the NEQ and PSQI are self-report instruments administered under similar conditions, which may have introduced common method bias. Furthermore, as the NEQ includes sleep-related items, some degree of conceptual overlap between the two instruments may exist. Therefore, the observed association between night eating behavior and sleep quality may partly reflect overlapping constructs or shared reporting characteristics rather than a relationship attributable solely to night eating behavior. Although the sensitivity analysis excluding sleep-related NEQ items partially addressed this concern, the modified score was exploratory and has not been independently validated. Therefore, the persistence of a statistically significant association should not be interpreted as eliminating measurement-related limitations. Another important limitation is that psychological distress, including depressive and anxiety symptoms, was not independently assessed in the present study. This is particularly relevant because the NEQ includes mood-related items, and psychological factors may be associated with both night eating-related symptoms and subjective sleep quality. In a study of Slovak university students, Falbová et al. [57] reported that sleep difficulties were strongly associated with lower mental well-being and higher depressive symptom levels, including among women. Although their study did not directly investigate night eating behavior, these findings highlight the importance of considering psychological health when interpreting sleep-related associations. Therefore, unmeasured psychological distress may have contributed to the observed relationship between NEQ and PSQI scores, and residual confounding cannot be excluded. Other potentially relevant factors, including shift work, chronotype, medication use, caffeine intake, and dietary habits, were not comprehensively assessed, while objective sleep measurements were not obtained. Furthermore, the recruitment of participants from multiple family health centers within a single district (Mamak, Ankara), together with the convenience sampling approach, may have introduced selection bias, as women attending these centers may differ from the general population of adult women. In addition, the large proportion of housewives or unemployed women and the predominance of participants who did not engage in regular exercise may further limit the generalizability of the findings to populations with different sociodemographic and lifestyle characteristics. Furthermore, regular exercise was assessed using a binary self-reported measure (yes/no), without applying a standardized definition based on exercise frequency, duration, or intensity. This approach may have resulted in misclassification and did not allow for a detailed assessment of physical activity levels. Therefore, the observed association between regular exercise and sleep quality should be interpreted with caution. Furthermore, menopausal status and reproductive history, including parity and miscarriage history, were not assessed in the present study. Given the wide age range of the participants (18–65 years), these unmeasured characteristics may be relevant to the interpretation of subjective sleep quality. Vorobeľová et al. [58] reported an association between miscarriage history and self-reported sleep disturbances among middle-aged women, highlighting the potential relevance of reproductive factors to sleep-related symptoms. Although their findings are not directly comparable to the present study, the absence of information on menopausal status and reproductive history represents an additional limitation, as their potential confounding effects could not be evaluated.

The strengths of the present study include its large sample size and the use of validated instruments to assess night eating behavior and sleep quality. In addition, the face-to-face administration of the questionnaire and study instruments by the researcher, as well as the fact that anthropometric measurements were obtained directly by the researcher rather than being self-reported, represent further strengths of the study. These measurements included waist circumference, waist-to-hip ratio, and waist-to-height ratio, providing additional indicators of adiposity beyond BMI.

## 5. Conclusions

In conclusion, higher night eating scores were moderately associated with poorer subjective sleep quality, whereas BMI showed weak positive correlations with both PSQI and NEQ scores. In the fully adjusted regression model, night eating behavior remained significantly associated with poorer sleep quality, while BMI was no longer statistically significant. Older age and current smoking were also associated with poorer sleep quality, whereas regular exercise was associated with better sleep quality. These findings suggest that night eating behavior is associated with subjective sleep quality in adult women, even after accounting for measured sociodemographic and lifestyle characteristics. However, the sensitivity analysis showed that the association was attenuated but remained statistically significant after excluding sleep-related NEQ items, highlighting the potential influence of overlapping questionnaire content on the original findings. Nevertheless, the cross-sectional design precludes causal interpretations, and the findings should be considered in light of potential residual confounding and measurement limitations. Future longitudinal and interventional studies are needed to clarify the direction of these associations.

## Figures and Tables

**Table 1:** Descriptive statistics for age, anthropometric measurements, sleep quality, and night eating scores of the participants (*n* = 1203).

Variable	Min–Max	Median	Mean ± SD
Age (years)	18.00–65.00	38.00	39.49 ± 11.22
Body weight (kg)	38.00–165.00	68.70	70.26 ± 14.10
Height (cm)	133.00–180.00	160.00	160.53 ± 6.28
Body mass index (kg/m^2^)	15.61–57.66	26.50	27.32 ± 5.63
Waist circumference (cm)	43.00–145.00	87.00	87.75 ± 14.33
Hip circumference (cm)	46.00–162.00	105.00	106.24 ± 11.73
Waist-to-hip ratio	0.39–1.34	0.81	0.82 ± 0.08
Waist-to-height ratio	0.28–0.87	0.54	0.54 ± 0.09
PSQI sleep disturbance score	0.00–3.00	1.00	1.32 ± 0.57
PSQI total score (7 components)	0.00–17.00	5.00	5.58 ± 2.84
Night Eating Questionnaire total score	1.00–39.00	12.00	12.50 ± 5.04

**Table 2:** Distribution of participants according to sociodemographic characteristics and lifestyle behaviors (*n* = 1203).

Variable	*n*	%
Marital status		
Married	941	78.2
Single	262	21.8
Education level		
Illiterate	32	2.7
Primary/secondary school	362	30.1
High school	445	37.0
University	326	27.1
Postgraduate (Master’s/PhD)	38	3.2
Occupation		
Student	67	5.6
Civil servant	127	10.6
Retired	33	2.7
Self-employed	75	6.2
Private sector employee	118	9.8
Housewife/Unemployed	781	64.9
Other	2	0.2
Smoking status		
Yes	308	25.6
No	860	71.5
Former smoker	35	2.9
Alcohol consumption		
Yes	101	8.4
No	1097	91.2
Former drinker	5	0.4
Regular exercise		
Yes	304	25.3
No	899	74.7

**Table 3:** Correlations among NEQ total score, PSQI total score, and BMI.

Variables	Correlation Coefficient	95% CI	*p*	*n*
NEQ total score-PSQI total score	ρ = 0.404	0.355 to 0.454	<0.001	1202
NEQ total score-BMI	ρ = 0.085	0.027 to 0.140	0.003	1202
PSQI total score-BMI	*r* = 0.122	0.065 to 0.179	<0.001	1203

Spearman’s rank correlation coefficient (ρ) was used for associations involving NEQ total score; Pearson’s correlation coefficient (*r*) was used for the association between PSQI total score and BMI. The 95% confidence intervals were estimated using 3000 bootstrap resamples. Higher PSQI scores indicate poorer subjective sleep quality. BMI, body mass index; NEQ, Night Eating Questionnaire; PSQI, Pittsburgh Sleep Quality Index.

**Table 4:** Hierarchical multiple linear regression models examining factors associated with PSQI total scores.

Model	Variables Entered	R^2^	Adjusted R^2^	F (df)	*p*	ΔR^2^	ΔF (df)	*p* for ΔF
1	NEQ total score	0.186	0.185	274.181 (1, 1200)	<0.001	0.186	274.181 (1, 1200)	<0.001
2	NEQ total score, BMI	0.195	0.194	145.349 (2, 1199)	<0.001	0.009	13.631 (1, 1199)	<0.001
3	NEQ, BMI, age, education, occupation, smoking, alcohol use, regular exercise	0.229	0.218	20.642 (17, 1184)	<0.001	0.033	3.426 (15, 1184)	<0.001

Note: PSQI, Pittsburgh Sleep Quality Index; NEQ, Night Eating Questionnaire; BMI, body mass index; R^2^, coefficient of determination; Adjusted R^2^, adjusted coefficient of determination; ΔR^2^, change in R^2^; F, overall model F-statistic; ΔF, F-change statistic; df, degrees of freedom. All regression models included 1202 participants.

**Table 5:** Regression coefficients of the fully adjusted model for PSQI total score.

Variable	B	HC3 SE	β	95% CI for B	*p*	Tolerance	VIF
Constant	0.404	0.515	-	−0.607 to 1.415	0.433	-	-
NEQ total score	0.240	0.016	0.425	0.208 to 0.272	<0.001	0.959	1.042
BMI	0.026	0.016	0.051	−0.006 to 0.058	0.115	0.768	1.303
Age (years)	0.031	0.008	0.122	0.015 to 0.047	<0.001	0.600	1.666
Education: illiterate	0.479	0.403	0.027	−0.312 to 1.269	0.235	0.885	1.131
Education: primary/middle school	0.320	0.199	0.052	−0.070 to 0.710	0.107	0.642	1.559
Education: university	−0.036	0.203	−0.006	−0.436 to 0.363	0.858	0.664	1.506
Education: postgraduate	−0.759	0.396	−0.047	−1.536 to 0.017	0.055	0.863	1.159
Occupation: student	0.564	0.360	0.045	−0.143 to 1.271	0.118	0.806	1.241
Occupation: civil servant	0.345	0.293	0.037	−0.230 to 0.919	0.240	0.755	1.325
Occupation: retired	0.397	0.544	0.023	−0.670 to 1.464	0.465	0.867	1.153
Occupation: self-employed	0.075	0.293	0.006	−0.501 to 0.650	0.799	0.950	1.053
Occupation: private sector	0.350	0.260	0.037	−0.161 to 0.860	0.179	0.867	1.153
Current smoker	0.563	0.190	0.086	0.191 to 0.936	0.003	0.854	1.171
Former smoker	0.625	0.463	0.037	−0.283 to 1.533	0.177	0.967	1.034
Current alcohol use	0.075	0.289	0.007	−0.491 to 0.641	0.795	0.857	1.167
Former alcohol use	0.440	1.123	0.010	−1.764 to 2.644	0.695	0.965	1.036
Regular exercise: yes	−0.397	0.165	−0.061	−0.721 to −0.074	0.016	0.980	1.021

Note: Dependent variable: PSQI total score. Results are from the fully adjusted hierarchical multiple linear regression model (Model 3; *n* = 1202). B, unstandardized regression coefficient; HC3 SE, heteroscedasticity-consistent standard error (HC3 method); β, standardized regression coefficient; CI, confidence interval; VIF, variance inflation factor; NEQ, Night Eating Questionnaire; BMI, body mass index; PSQI, Pittsburgh Sleep Quality Index.

**Table 6:** Comparison of anthropometric measurements according to PSQI sleep quality classification.

	PSQI Sleep Quality Classification		
	Good Sleep Quality (≤5) (*n* = 663)	Poor Sleep Quality (>5) (*n* = 540)	
	Mean ± SD	Mean ± SD	*p*-Value
Body weight (kg)	69.15 ± 13.04	71.63 ± 15.20	0.003
Height (cm)	160.92 ± 6.23	160.07 ± 6.32	0.020
BMI (kg/m^2^)	26.76 ± 5.19	28.03 ± 6.06	<0.001
Waist circumference (cm)	86.18 ± 13.45	89.68 ± 15.12	<0.001
Hip circumference (cm)	105.25 ± 11.50	107.46 ± 11.89	0.001
Waist-to-hip ratio	0.81 ± 0.08	0.82 ± 0.09	0.006
Waist-to-height ratio	0.53 ± 0.08	0.55 ± 0.10	<0.001

Data are presented as mean ± standard deviation (SD). Between-group comparisons were performed using independent-samples *t*-tests; Welch’s correction was applied when the assumption of homogeneity of variances was not met. All *p*-values are two-sided. PSQI, Pittsburgh Sleep Quality Index. PSQI scores ≤ 5 indicate good sleep quality, whereas scores > 5 indicate poor sleep quality.

**Table 7:** Comparison of Night Eating Questionnaire total scores according to PSQI sleep quality classification.

Variable	Good Sleep Quality (PSQI ≤ 5), *n* = 663	Poor Sleep Quality (PSQI > 5), *n* = 539	Mann–Whitney *U*	*Z*	*p*	*r*
NEQ total score, median [Q1–Q3]; mean rank	11 [8,9,10,11,12,13]; 498.61	13 [11,12,13,14,15,16,17]; 728.06	110,461.5	−11.427	<0.001	−0.330

Note: Values are presented as median [Q1–Q3] and mean rank. The IQR was 5 in the good-sleep group and 6 in the poor-sleep group. Although 540 participants were classified as having poor sleep quality, one had missing NEQ data; therefore, the NEQ analysis included 539 participants in this group (*N* = 1202 overall). The effect size was calculated as *r* = Z/√*N*. PSQI, Pittsburgh Sleep Quality Index; NEQ, Night Eating Questionnaire; IQR, interquartile range.

## Data Availability

The data supporting the findings of this study are available from the corresponding author upon reasonable request. The data are not publicly available due to privacy and confidentiality considerations concerning participant information.

## Abbreviations

The following abbreviations are used in this manuscript:

BMI	Body Mass Index
HPA	Hypothalamic–Pituitary–Adrenal
NEQ	Night Eating Questionnaire
NES	Night Eating Syndrome
PSQI	Pittsburgh Sleep Quality Index
SD	Standard Deviation
VIF	Variance Inflation Factor
